# Supplementary material for: Resolving the full spectrum of human genome variation using Linked-Reads
Source: Genome Res. 2019 Apr;29(4):635–45. doi: 10.1101/gr.234443.118 (PMC6442396; doi:10.1101/gr.234443.118)
Supplement: Supplemental Material [file supp_gr.234443.118_Supplemental_longranger-2.2.zip › longranger-2.2.tar.bz2!/lr2.2-public/lib/stan/cmdstan/stan/doc/api/html/structstan_1_1io_1_1stan__csv__timing.html]

Stan: stan::io::stan\_csv\_timing Struct Reference


|  |  |
| --- | --- |
|  | Stan  2.5.0  probability, sampling & optimization |


- Main Page
- Related Pages
- Namespaces
- Classes
- Files

- Class List
- Class Index
- Class Hierarchy
- Class Members

All Classes Namespaces Files Functions Variables Typedefs Enumerations Enumerator Friends Macros Pages

- stan
- io
- stan\_csv\_timing

Public Member Functions |
Public Attributes |
List of all members

stan::io::stan\_csv\_timing Struct Reference

`#include <stan_csv_reader.hpp>`

|  |  |
| --- | --- |
| Public Member Functions | |
|  | stan\_csv\_timing () |
|  | |

|  |  |
| --- | --- |
| Public Attributes | |
| double | warmup |
|  | |
| double | sampling |
|  | |

## Detailed Description

Definition at line 52 of file stan\_csv\_reader.hpp.

## Constructor & Destructor Documentation

|  |  |  |  |  |
| --- | --- | --- | --- | --- |
| stan::io::stan\_csv\_timing::stan\_csv\_timing | ( |  | ) |  |

inline

Definition at line 56 of file stan\_csv\_reader.hpp.

## Member Data Documentation

double stan::io::stan\_csv\_timing::sampling

Definition at line 54 of file stan\_csv\_reader.hpp.

double stan::io::stan\_csv\_timing::warmup

Definition at line 53 of file stan\_csv\_reader.hpp.

---

The documentation for this struct was generated from the following file:

- src/stan/io/stan\_csv\_reader.hpp


---

[ Stan Home Page ]

*© 2011–2014,
Stan Development Team.*
